# Pump‐Color Selective Control of Ultrafast All‐Optical Switching Dynamics in Metaphotonic Devices

**DOI:** 10.1002/advs.202000799

**Published:** 2020-06-05

**Authors:** Yuze Hu, Jie You, Mingyu Tong, Xin Zheng, Zhongjie Xu, Xiangai Cheng, Tian Jiang

**Affiliations:** ^1^ College of Advanced Interdisciplinary Studies National University of Defense Technology Changsha 410073 P. R. China; ^2^ National Innovation Institute of Defense Technology Beijing 100010 P. R. China

**Keywords:** electromagnetically induced transparency, germanium, silicon, terahertz metamaterials, ultrafast photoswitching

## Abstract

Incorporating active materials into metamaterials is expected to yield exciting advancements in the unprecedented versatility of dynamically controlling optical properties, which sheds new light on the future optoelectronics. The exploration of emerging semiconductors into terahertz (THz) meta‐atoms potentially allows achieving ultrafast nanodevices driven by various applications, such as biomedical sensing/imaging, ultrawide‐band communications and security scanners. However, ultrafast optical switching of THz radiation is currently limited to a single level of tuning speed, which is a main hurdle to achieve multifunctionalities. Here, a hybrid metadevice which can realize the pump‐wavelength controlled ultrafast switching response by the functionalization of double photoactive layers is demonstrated experimentally. A whole cycle of electromagnetically induced transparency switching with a half‐recovery state changes from 0.78 ns to 8.8 ps as pump wavelength varies from near infrared to near ultraviolet regions. The observed pump‐color selective switching speed changing from nanosecond scale to picosecond scale is ascribed to the wavelength‐dependent penetration length of Ge and the contrasting defect states between noncrystalline Ge and epitaxial Si layers. It is believed that the schemes regarding pump‐color controllable ultrafast switching behavior introduced here can inspire more innovations across the field of ultrafast photonics and can boost the reconfigurable metamaterial applications.

## Graphical abstract

All‐optical terahertz modulators with pump‐color selective ultrafast switching speed are realized by combining double layers of semiconductors with electromagnetically induced transparency meta‐atoms. The transmission/group delay of terahertz wave is modulated at contrasting switching speeds from sub‐nanoseconds to picoseconds when pumped by the near‐infrared and near‐ultraviolet beams. This work opens up new avenues for manipulating the dynamics of ultrafast metaphotonic devices.

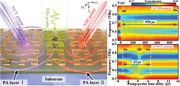

## 1 Introduction

Tunable metasurfaces have established as an interesting and intriguing research topic that attracts abundant of investigations and implementations in the field of optics.^[^ ^1^, ^2^, ^3^, ^4^ ^]^ As a 2D counterpart of metamaterials, metasurfaces studies are driven by the desire to efficiently tailor a large number of exotic electromagnetic responses with unprecedented properties in naturally existing media.^[^ ^5^ ^]^ The intense light matter interactions between functional unit meta‐atoms and incoming incidence have induced numerous novel phenomena, including anomalous refraction,^[^ ^6^, ^7^ ^]^ super lenses,^[^ ^8^, ^9^, ^10^ ^]^ invisibility cloaking,^[^ ^11^ ^]^ and polarization controlling,^[^ ^12^ ^]^ etc. In order to promote its real‐world applications, continuous attempts have been made to dynamically control the resonant properties of terahertz (THz) metasurfaces in a real‐time operation by executing external stimuli, including the optical pump,^[^ ^13^, ^14^, ^15^, ^16^, ^17^, ^18^, ^19^, ^20^, ^21^, ^22^, ^23^, ^24^, ^25^, ^26^, ^27^, ^28^, ^29^ ^]^ temperature change,^[^ ^30^, ^31^, ^32^, ^33^, ^34^, ^35^, ^36^ ^]^ mechanical extension,^[^ ^37^, ^38^, ^39^, ^40^, ^41^, ^42^ ^]^ and voltage/current bias.^[^ ^43^, ^44^, ^45^, ^46^, ^47^, ^48^, ^49^, ^50^, ^51^, ^52^, ^53^, ^54^, ^55^, ^56^ ^]^ Among which, the integration of photoactive semiconductors into THz meta‐atoms offers a feasible approach to realize an ultrafast switching speed, which can be ascribed to the fast relaxation dynamics of photoexcited carriers. Considering the different characteristics of light response, numerous active materials have recently been proposed to tailor THz waves, which encompass the conventional semiconductors,^[^ ^22^, ^23^, ^24^, ^57^, ^58^, ^59^, ^60^, ^61^, ^62^, ^63^, ^64^ ^]^ perovskites,^[^ ^25^, ^65^, ^66^ ^]^ superconductors,^[^ ^67^ ^]^ transition metal dichalcogenides,^[^ ^68^, ^69^ ^]^ topological insulators,^[^ ^70^ ^]^ graphene‐Si heterostructures,^[^ ^48^, ^71^, ^72^ ^]^ and Weyl semimetals.^[^ ^73^ ^]^ Especially for the applications of all‐optical metaphotonic devices, their vital figure‐of‐merits are closely related to photocarrier dynamics. Therefore, the ability to actively tune the ultrafast switching speed should be further advanced, opening up a new prospect for the areas of science and engineering. Unfortunately, according to the experiments reported so far, a robust approach that can tailor the ultrafast dynamics of metadevices in a simple, economy, easily operated approach is still missing.

One of the great challenges in addressing this issue is that the optical‐induced free carrier dynamics in the semiconductors are difficult to be altered during the post‐growth process. Therefore, attempts have been made to control the dynamics of the ultrafast semiconductor‐hybrid metaphotonic devices by varying the photoactive media that determine the device ultrafast performance, which can change from nanoseconds to picoseconds. As two representative materials in modern optoelectronics, silicon (Si) and germanium (Ge) have recently been introduced into the THz actively tuning platforms, possessing a natural compatibility with commercially used complementary metal‐oxide‐semiconductor (CMOS) technology.^[^ ^60^, ^61^, ^74^ ^]^ A prominent advantage of these two semiconducting materials is that their photocarrier lifetimes can be readily shortened from several milliseconds to picoseconds when the lattice defects are induced through the ion implantation, interfacial engineering or low‐temperature growth.^[^ ^75^, ^76^, ^77^ ^]^ However, it is not possible to trigger the transient dynamic transitions by either adopting a novel type of semiconductor or inserting lattice defects, once the hybrid device is fabricated. Consequently, a novel mechanism of manipulating the switching dynamics is urgently needed. One remarkable feature of semiconductors is the wavelength‐dependent absorption, resulting in a contrast of penetration length when illuminated by different colors of light, which has yet been exploited to manipulate the transient dynamics in ultrafast photonics. Ge provides itself great potentials in this application, since it possesses distinguish absorption coefficients in the ultraviolet–visible–infrared (UV–vis–IR) range. The penetration length varies from tens of nanometers to hundreds of nanometers when illuminated by both edges of the visible waveband. In this regard, it turns out that the amount of photocarriers in Ge layer can be arbitrarily steered by the change in pump wavelength. Thus, it raises an important question whether we can cleverly design an integrated photoactive layer that harvests the unabsorbed light in Ge film and then the photocarriers excited by the penetrated light relax with a contrastingly different lifetime.

In this work, we comprehensively investigate the pump‐wavelength controlled ultrafast switching response of a hybrid metadevice. Specifically, a Ge film of 120 nm is thermally evaporated onto epitaxial Si (500 nm) hybrid electromagnetically induced transparency (EIT) meta‐atoms to form an integrated photoactive layer. The bottom Si film with much smaller density of trap‐assisted recombination sites can absorb the light penetrating through the Ge film. The density of free carriers in both layers can be arbitrarily controlled by changing the wavelength of optical pump beam, thereby showing an ultrafast switching behavior that is sensitive to pump color with a high dynamic range, from sub‐nanoseconds to picoseconds. In particular, the near‐infrared (NIR) pump almost penetrates the Ge film and excites the free carriers in Si layer, giving rise to a half‐recovery time of normalized transmission resonance modulation 0.78 ns with a group delay modulation up to 3.3 ps. When pumped by near‐ultraviolet (NUV) light, only the upper Ge film is excited, and a half‐recovery cycle of EIT effect is accomplished as fast as 8.8 ps, corresponding to a group delay modulation up to 3.3 ps. This work makes possible for the dynamical tuning of ultrafast switching speed in metaphotonic devices hybrid with semiconductors, which may open a fascinating direction that affords great design flexibility for ultrafast optoelectronics.

## 2 Results and Discussion

### 2.1 Hybrid Meta‐Atoms Design and Characterization

**Figure 1:**
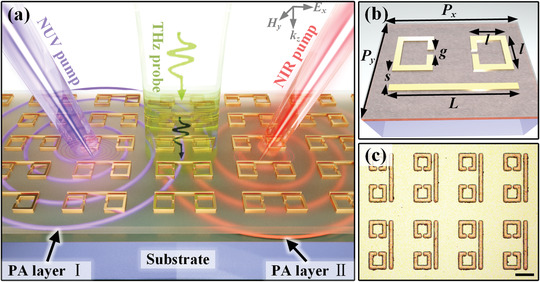
Design principle and characterization of double PAL hybrid meta‐atoms. a) Graphical architecture of proposed metaphotonic device functionalized with pumping wavelength‐controlled switching speed, which consists of two semiconductor layers, with the upper layer exhibiting a highly wavelength‐dependent penetration depth. The NUV pump merely hits the PAL I whereas the NIR pump penetrates through PAL I, thereby, exciting layer II with remarkable different free carriers’ relaxation time. The THz wave propagating along the *k_z_* direction is polarized along *E_x_* and impinges on the device normally. b) A unit cell of the meta‐atoms with the geometrical parameters given by *L* = 85 µm, *l* = 28 µm, *s* = 5 µm, *g* = 5 µm, *P_x_* = 80 µm, and *P_y_* = 120 µm. Here the thicknesses of sapphire substrate, silicon layer, and germanium layer are 500 µm, 500 nm, and 120 nm, respectively. c) Optical microscope image of the fabricated hybrid meta‐atoms. Scale bar, 20 µm.

Working principle of the hybrid meta‐atoms functionalized by the pumping wavelength‐controlled switching response (PWCS‐MM) is schematized in **Figure** **1**a. Each functional cell of the proposed PWCS‐MM employs a well‐studied EIT metamaterial consisting a cut‐wire (CW) surrounded by two split ring resonators (SRRs), whose subwavelength sizes are carefully optimized to ensure a Fano‐type linear destructive interference. When under a photoexcitation, such Fano‐type meta‐atoms integrated with semiconductors can exhibit an active switching feature at a given switching speed that is dependent on the carriers’ relaxation time. Therefore, the key to our expected functionality is the clever construction of multiple semiconductors with different photocarriers decay rates. Briefly, by subtly taking advantage of the optical penetrating length difference of two semiconductors, our proposed scheme can be described as follows: a) the near‐ultraviolet pumping beam merely excite the free carriers in the upper photon absorption layer (PAL I); b) the near‐infrared pumping beam passes through PAL I until it is absorbed by the bottom photon absorption layer (PAL II) with a contrasting photocarriers relaxation rate. Considering the above prerequisites, the 120 nm thermal evaporation germanium (Ge) film is adopted as PAL I, whereas the 500 nm silicon (Si) naturally epitaxial grown on the sapphire substrate is selected as PAL II. For a better illustration, the thicknesses of both layers in Figure 1a are purposely increased and the corresponding interactions with the pumping beams of different wavelengths are vividly illustrated. The first step of the fabrication process for the MMs is the deposition of an Au layer on the PAL II with the thickness of 150 nm, followed by a thermal evaporation of PAL I. The frequency independent conductivity of the gold can be assumed to be 4.56 × 10^7^ S m^−1^ in the THz regime. The incident THz wave is polarized along the CW‐aligned (designated as *x*‐) direction and normally propagated along the *k_z_* direction.

Figure 1b demonstrates the geometric shape of the appropriately designed MMs within an individual cell that exhibits a distinguished EIT peak. The MMs consist of a pair of SRRs attached to a CW and the vital geometrical parameters are given below: the CW is aligned along *x*‐direction, whose length is selected to be *L* = 85 µm, in order to generate a broad background dipole (DP) resonance; the width of the SRRs is *l* = 28 µm; the distance between the CW and SRRs is *s* = 5 µm, allowing for the interaction between each other; the gap length of SRRs acting as a capacitor to support an inductive–capacitive (LC) resonance is *g* = 5 µm. The optical microscope image of fabricated meta‐atoms is shown in Figure 1c, from which we could distinguish the meta‐atoms clearly.

### 2.2 Photoexcited EIT Switching of PWCS‐MM

**Figure 2:**
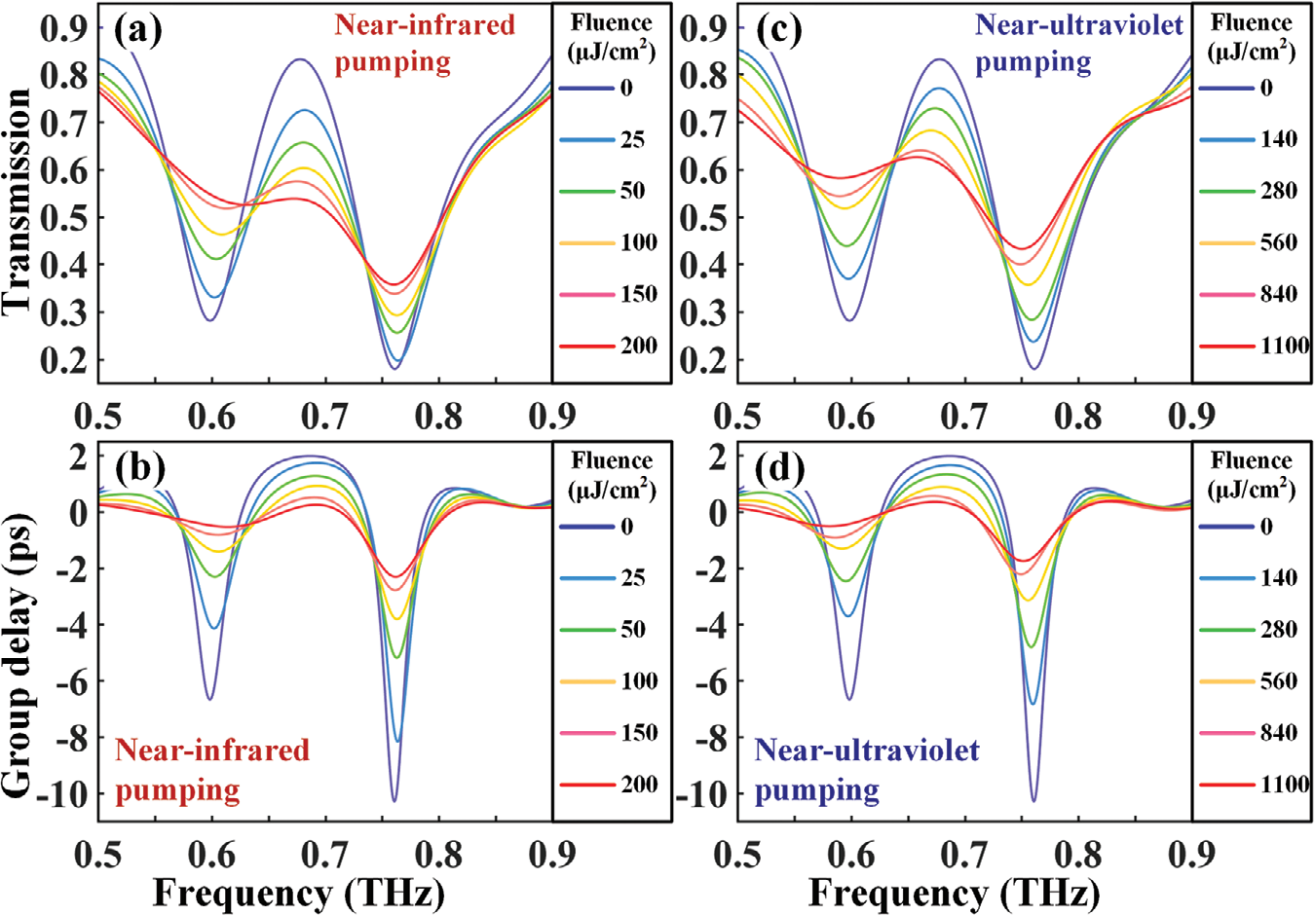
Fluence‐dependent modulation of the EIT resonances in double‐PA hybrid meta‐atoms. a,b) THz transmission and group delay spectra measured at various pump fluences from 0 µJ cm^−2^ to 200 µJ cm^−2^, under the NIR pumping condition of 800 nm. c,d) THz transmission and group delay spectra measured at various pump fluences from 0 to 1100 µJ cm^−2^, under the NUV pumping condition of 400 nm.

In order to methodically demonstrate the optical properties of PWCS‐MM, we record the terahertz transmission and group delay spectra, accounting for different fluences of both NUV and NIR pump pulses, with the results shown in **Figure** **2**. From Figure 2a,c, it is easy to discern a strong EIT resonance feature shown as the blue curve in the THz transmission spectra. The DP mode induced by the CW has a quality (*Q*) factor of ≈2.8 centered at 0.7 THz, and the LC resonance of SRR‐pair exhibits a strong EIT window with a *Q* factor ≈6.5 at 0.65 THz. It is exceedingly necessary to stress that the sharp transparency peak is caused by the near field coupling between the DP and LC modes, and the LC resonance is more susceptible to changes in its surrounding environment. Consequently, the annihilation of the Fano line‐shaped resonance is more obvious than the suppression of DP resonance when the photoinduced conductivity short‐circuits the gap split in of the SRRs, thus leading to a strong modulation of transmission amplitude as well as the dispersion of group delay spectrum. By defining the EIT sharp transmission resonance strength as *δT* = *T*_EIT peak_ − *T*_EIT dip_ with the peak at 0.65 THz and dip at 0.6 THz, the EIT transmission resonance strength without photoexcitation is extracted to be as high as 54%. Once the 800 nm (NIR) optical pump beam photoexcites the PWCS‐MM sample, a strong reduction in the amplitude of the EIT sharp is observed and the saturable trend is found, as shown in Figure 2a. When the fluence increases up to 200 µJ cm^−2^, the EIT resonance is completely switched‐off with *δT* = 0. We subsequently investigate the transmission modulation under a 400 nm (NUV) excitation but with a higher pump fluence, aiming at realizing a same degree of switching‐off level. In experimental measurement, the pump‐probe time delay is matched at the maximal THz response state. The upgraded level of pump fluence can be ascribed to two possible reasons. First, the photoconductivity of the amorous Ge is less than the epitaxial Si. Next, the exceedingly fast relaxation rate in Ge layer leads to a decay time much shorter than the THz pulse profile (please see Figure S1, Supporting Information), resulting in an insufficient interaction time between the THz pulse and short‐circuited SRRs. The EIT transmission resonance strength *δT* is changed from 54% to 3% with the fluence varying from 0 to 1100 µJ cm^−2^, which is also effectively switched‐off.

Another intriguing phenomenon associated with strong dispersions of EIT transmission amplitude spectra is the slow light behavior. The optically active slow light performance is analyzed by retrieving from the group delay definition Δ*t*_g_ = −d*ϕ*/d*ω*, with *ϕ* being the phase and *ω =* 2*πf* being the angular frequency. The measured spectra in Δ*t*_g_ as a function of the pump fluence are plotted in Figure 2b,d for NIR and NUV pumping conditions, respectively. With the resonance strength of EIT group delay being denoted as *δt*_g_ = Δ*t*_g EIT peak_ − Δ*t*_g EIT dip_, the hybrid MMs give rise to an EIT group delay resonance strength *δt*_g_ up to 8.8 ps. The strength tends to be suppressed upon the increment of fluence, which is saturated at *δt*_g_ ≈ 0 in case of 200 µJ cm^−2^ for NIR or 1100 µJ cm^−2^ for NUV. In order to further verify the assumption that above‐mentioned phenomena stem exclusively from the variation of photoconductivity, we numerically simulated the transmission and delay spectra by changing the conductivity of PALs I and II, corresponding to the cases of Figure 2, with the new results presented in Figure S2 (Supporting Information). The slight discrepancy between the measured data and simulated results may be caused by the mismatches from fabrication. Notably, to achieve a similar degree of switching, a roughly fourfold amount of conductivity in the Ge layer is needed when compared to that of the Si layer, which is ascribed to the thickness difference between two layers. However, the pump fluence of NUV is nearly six times higher than that of the NIR case, which further supports the claim that shorter carriers decay rate and low photoconductivity of evaporated Ge result in slighter modulation depth.

### 2.3 Pump Wavelength‐Controlled Switching Dynamics

**Figure 3:**
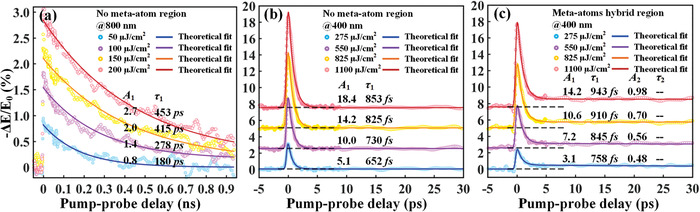
Pumping wavelength‐dependent relaxation dynamics of the double‐PA and the hybrid metaphotonic device. a) Negative differential transmission measured at a selection of pump fluences versus optical‐pump THz‐probe time delay, pumped by an NIR 800 nm optical pulse. Negative differential transmission measured at a selection of pump fluences pumped by an NUV 400 nm optical pulse in the double‐PA regions, b) with and c) without meta‐atoms, respectively.

Before proceeding the thorough discussion of the core functionality of wavelength‐controlled switching dynamics, we first characterize the time‐resolved, wavelength‐dependent THz conductivity in double PALs by performing the optical‐pump THz probe (OPTP) measurements. Hence, we can meticulously inspect the general transient charge‐carrier dynamics in our proposed integrated semiconductor film excited by either NIR or NUV pump. **Figure** **3** demonstrates the differential transmission (−∆*E*/*E*_0_) of the terahertz pulse at different pump fluences and wavelengths. When the integrated PAL pumped by the NIR beam of 800 nm, we can extract the lifetime of the decay process, all transients are fitted by an exponential decay function given by

(1) $$\begin{array}{c} -\Delta E/E_{0}=A_{0}+A_{1}e^{\frac{t-t_{0}}{\tau_{1}}} \end{array}$$

where *A*_0_ is a constant signal, *A*_1_ represents the weight of the exponential decay process, *τ*_1_ is responsible for the lifetime of photocarriers, and *t*_0_ is a time constant that determines the start time of decay fitting. Relaxation dynamics of PAL II (silicon epilayer) are clearly shown in Figure 3a, which are found to increase from 180 to 453 ps as the pump fluence changes from 50 to 200 µJ cm^−2^. The retrieved dynamics constitute a direct manifestation of a fact that the NIR‐pump pulse can mainly penetrate through the PAL I and induce the photocarriers in PAL II where a typical slow recombination rate of a silicon epilayer is discovered. Next, we turn to the investigation of the photocarrier dynamics pumped by the NUV beam. Since the relative change in THz transmission induced by quasi‐metal transition of thermally sputtered Ge generally possesses the sub‐picosecond decay time constants, the convolution of the exponential decay profiles and the instrument response function (IRF) are needed to fit the experimentally measured data points, which is mathematically expressed as

(2) $$\begin{array}{c} -\Delta E/E_{0}=e^{-{\left(\frac{t-t_{0}}{\mathrm{IRF}/2\ln2}\right)}^{2}}\times \left(A_{0}+A_{1}e^{-\frac{t-t_{0}}{\tau_{1}}}\right) \end{array}$$

where the IRF is the width of the instrument response function, relative to the full‐width at half‐maximum of the pump pulse. From the experimental results in Figure 3b, the decay time of theoretical fitting curves exhibits a monotonic increase from 652 fs (at 275 µJ cm^−2^) to 853 fs (at 1100 µJ cm^−2^), which suggests an ultrafast recombination rate that is improved by two orders of magnitude compared to that pumped by the NIR beam. The monitored phenomenon unambiguously shows that the NUV cannot penetrate the PAL I (i.e., noncrystalline Ge), which plays an important role in the wavelength‐controlled switching speed. The increment of decay time as a function of pump fluence is mainly attributed to the limited available trap‐assisted recombination sites resulted from defect states. As large number of photoexcited free carriers could overpopulate the recombination sites, the decay rate slows down time to increase the decay time with higher pump fluence. In order to confirm whether this scheme is suitable for the hybrid meta‐atoms case, it is extremely necessary to explore the photocarrier dynamics induced by NUV pump at the meta‐atom hybrid region. The most striking feature in Figure 3c is that the recombination process cannot be described by a mono‐exponential decay, and the slow exponential decay part should be added, which is given by

(3) $$\begin{array}{c} -\Delta E/E_{0}=e^{-{\left(\frac{t-t_{0}}{\mathrm{IRF}/2\ln2}\right)}^{2}}\times \left(A_{0}+A_{1}e^{-\frac{t-t_{0}}{\tau_{1}}}+A_{2}e^{-\frac{t-t_{0}}{\tau_{2}}}\right) \end{array}$$

with the added items *A*_2_ and *τ*_2_ corresponding to the slow recombination process. As illustrated in Figure 3c, in addition to the ultrafast dynamics at sub‐picosecond scales determined by PAL I, the slow relaxation process in PAL II also occupies a relatively small weight, which is beyond our expectations. Since the photoconductivity of epitaxial Si is much higher than that of the Ge film, the leakage of photons into the Si layer is severely detrimental to the high switching ratio under the NUV pump. At this point, it is important to stress that the PAL I is evaporated after the meta‐atoms are fabricated. Hence, a possible reason behind is the invisible gaps between the Ge film and the edges of meta‐atoms existing in the fabrication process. A small amount of scattered NUV pump light penetrates the small gaps and thus excites the Si epilayer, leading to a no‐full recovery after the sub‐picosecond relaxations. One potential solution is to optimize the processing technology, such as using a negative type photoresist, which is beyond the scope of our discussion. As abovementioned, we can simply alter the ultrafast EIT modulation speeds for both timescales by changing the optical pump wavelength.

#### 2.3.1 Functionality I: Nanosecond‐Scaled EIT Switching Pumped by Near‐Infrared Beam

**Figure 4:**
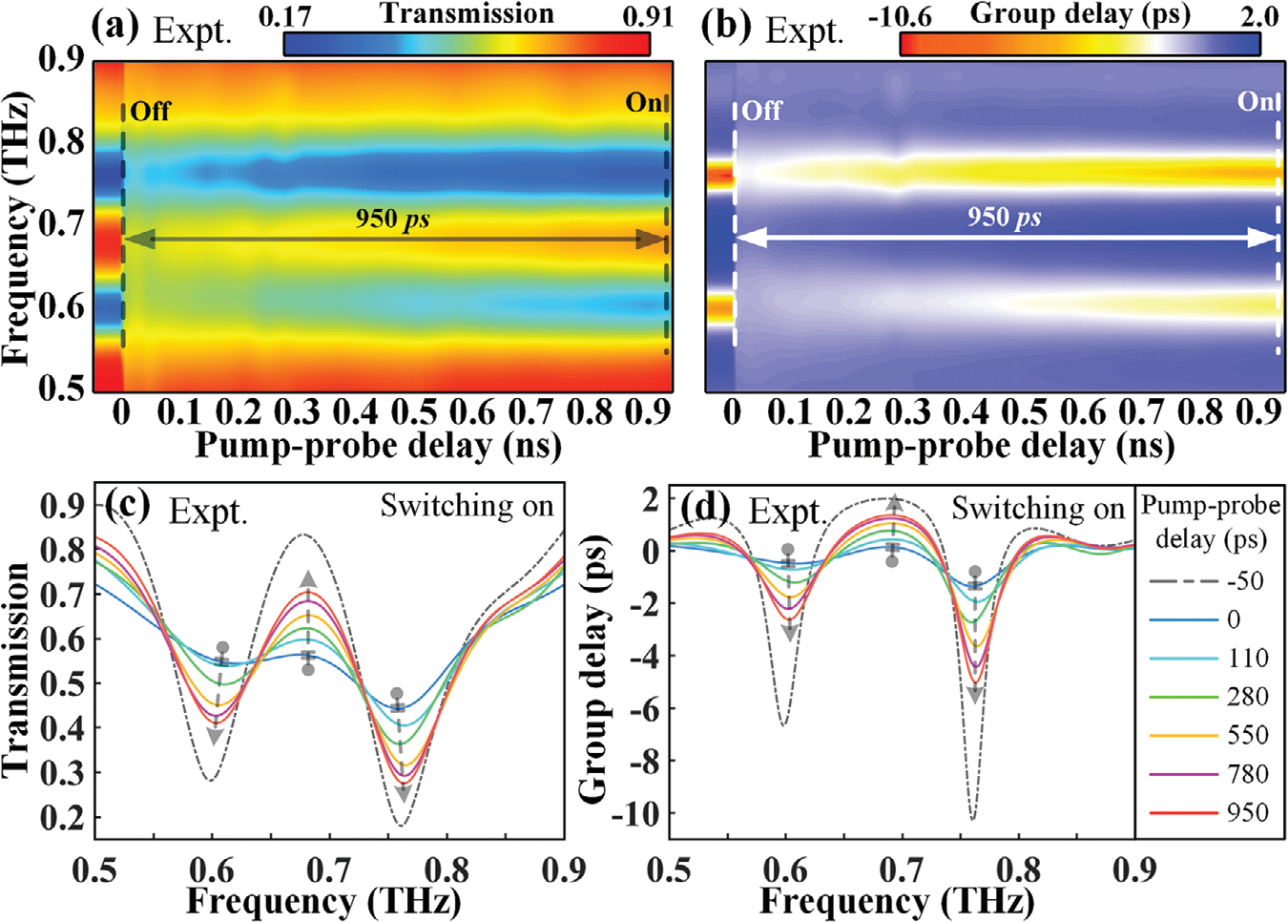
NIR‐pumped transient transmission and group delay switching of the double‐PA hybrid meta‐atoms. a,b) Experimentally measured color maps against optical‐pump THz‐probe delay for transmission and group delay switching dynamics, respectively. c,d) Corresponding transient spectra at various pump‐probe time delays extracted from the maps of a) and b), respectively. The pump fluence is 200 µJ cm^−2^. The arrowheads between the dashed lines indicate the half‐recovery time of ≈1 ns for the transmission modulation, showing a relative slow switching speed.

To determine the device performance, we first explore the optically tunable terahertz response of the double‐PAL hybrid metaphotonic device when injected by NIR pumps. **Figure** **4** illustrates the switching dynamics of the THz transmission amplitude and group delay spectra under an 800 nm pump with a fluence of 200 µJ cm^−2^. The time delay here is identified as the time difference between the arrival of the optical pump pulse and the terahertz probe pulse at the surface of sample, and the maximal THz modulation response is selected at the time delay of 0 ps. As visualized in Figure 4a, when the NIR femtosecond pump pulse is incident on the PALs at 0 ns, the EIT resonance switches from on‐state to off‐state immediately, owing to a large number of free carriers photoexcited in the PAL II. Then, a complete switched‐off state with *δT* = 0 tends to recover to the on state with *δT* = 54% at a relatively slow speed and with the increment of time delay. It is clear seen that the EIT resonance is not fully recovered even at the maximal pump‐probe time delay (0.95 ns), limited by our measurement system. As a physically pertinent phenomenon, the transient dynamic of group delay is visualized in Figure 4b. It turns out that it undergoes a similar transient evolution dynamics when compared to the transmission spectra, since the photocarriers have the same relaxation process. This observation unequivocally shows that the active tuning dynamics of hybrid metadevice in the NIR‐pumped case relies on the photoconductivity in PAL II, allowing for a nanosecond timescaled EIT switching response.

The spectra extracted from the false‐color maps as a function of time delay allows for the exploration of the features of functionality I, as shown in Figure 4c,d. For the transmission spectra in Figure 4c, it is easy to discern that the resonance strength recovers from 0% at 0 ps to 29% at 950 ps. Normally to retrieve the extent of switching‐off state, we define the normalized resonance strength of THz transmission as *R*_*δ*_ *_T_* = (*δT*_‐5 ps_ − *δT*)/*δT*_‐5 ps_, where *δT* is the EIT transmission resonance strength at a given pump‐probe time delay. Following this definition, *R*_*δ*_ *_T_* = 1 at 0 ps represents a full annihilation of EIT resonance. As we shall see, the time delay of 780 ps is *R*_*δ*_ *_T_* = 0.5, corresponding to a half‐recovery time constant. In addition, the sharp spikes in Figure 4d present the group delay recovery process, with the group delay modulation up to 3.3 ps for the EIT group delay resonance strength at 0.6 THz, which is within the period of the half‐recovery time 780 ps. Herein, pumped by an NIR beam, we can reiterate the triumph of realizing an EIT switching on nanosecond scale with a half‐recovery time 780 ps.

#### 2.3.2 Functionality II: Picosecond‐Scaled EIT Switching Pumped by Near‐Ultraviolet Beam

**Figure 5:**
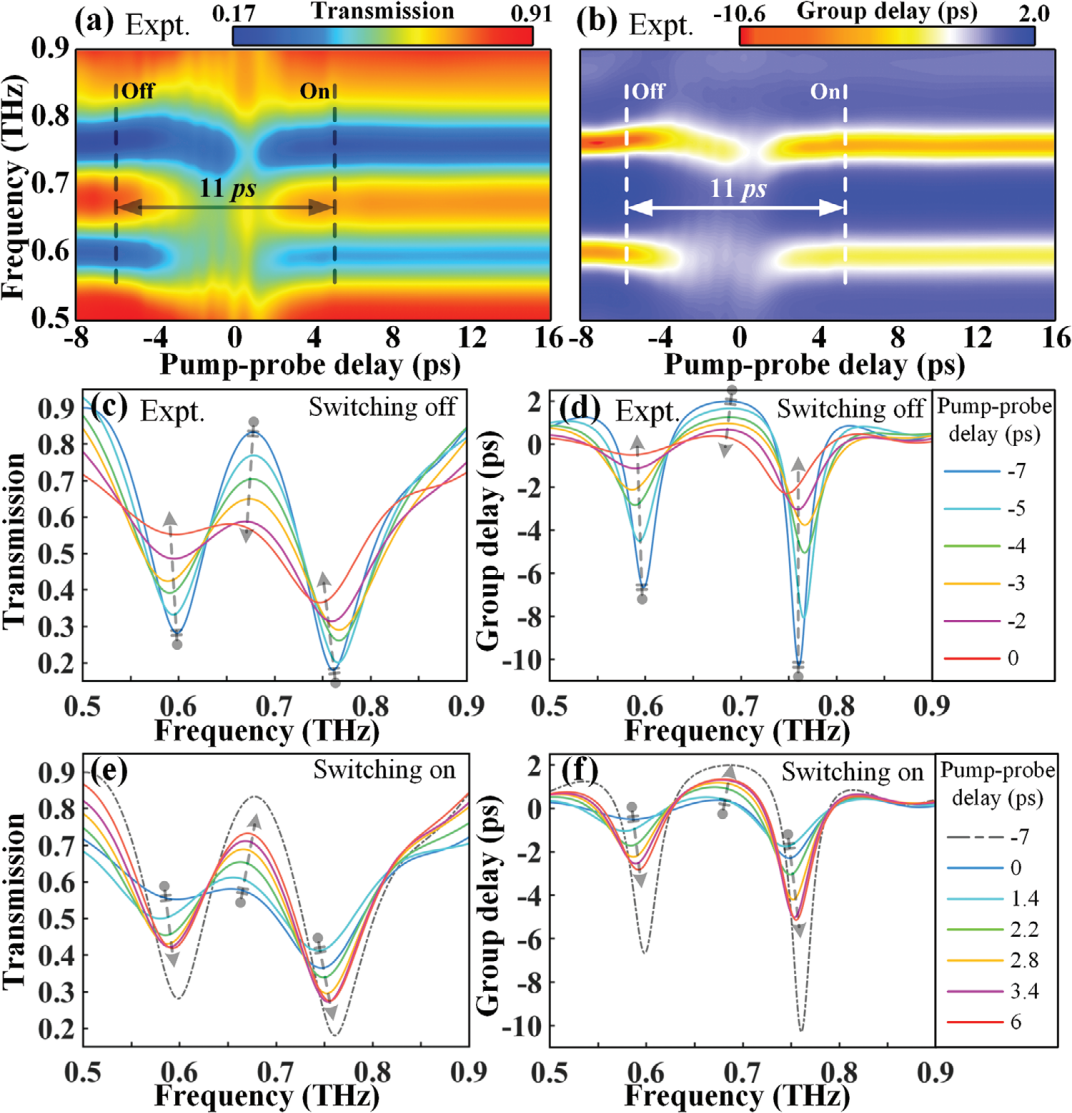
NUV‐pumped transient EIT switching of the double‐PA hybrid meta‐atoms. a,b) Experimentally measured color maps against optical‐pump THz‐probe delay for transmission and group delay switching dynamics, respectively. c,d) Corresponding transient spectra at various pump‐probe time delays extracted from the maps of (a) and (b), respectively. The pump fluence is fixed at 1100 µJ cm^−2^. The arrowheads between the dashed lines indicate the half‐recovery time ≈10 ps for the transmission modulation, indicating that the switching speed is two orders of magnitude faster compared with the case under NIR pump.

The ultrafast all‐optical switching dynamics at a picosecond scale and under the photoexcitation of the NUV pump is comparatively studied in this section. The temporal evolutions of the THz transmission and group delay spectra are depicted in the false‐color maps in Figure 5a,b, respectively. On the first glance, surprisingly, the whole cycle of switching‐off and ‐on processes is as fast as the timescale of 11 ps, laying a foundation for one to control the ultrafast dynamics of metaphotonic devices by a simple pump wavelength alternation. Precisely, the EIT effect of double‐PAL hybrid meta‐atoms is completely switched off within 6 ps, and the recovery time is ≈5 ps, but without a full recovery rate remaining essentially unchanged after a 5 ps time delay. The results directly associate with the intrinsic nature of the ultrafast relaxation dynamic in noncrystalline Ge. The phenomenon that the EIT‐resonance is not well switched‐on is also mainly due to the leaky NUV pump light into PAL II. From an alternate perspective, the photoconductivity contribution in PAL I is much larger than that in PAL II, thus resulting in a picosecond timescaled EIT switching.

To quantitatively characterize the working efficiency of the proposed metaphotonic devices operating at a picosecond timescale, we clearly show the spectra against the time delay from the maps of Figure 5a,b. The switching‐off evolutions for transmission amplitude and group delay are unambiguously demonstrated in Figure 5c,d, respectively. The EIT effect at −7 ps (*R*_*δ*_ *_T_* = 1, *δt*_g_ = 8.8 ps) emerges with an ultrafast switching‐off speed, which is totally compressed at 0 ps (*R*_*δ*_ *_T_* = 0, *δt*_g_ = 0 ps). In view of the relaxation lifetime that is much shorter than the THz probe pulse, the switching‐off time period can be identified as the time segment for the excited photocarriers to interact with the THz probe pulse before overlapped at the moment of the THz pulse peak. After 0 ps, the temporal evolutions toward steady (on) state of the THz spectra under the influence of the NUV optical pump are quantified in Figure 5e,f. With the increment of time delay, the saturable trend toward the steady state (red curves in Figure 5e,f) is obvious and the modulation depth can be viewed as almost a constant after 3.4 ps. With the definition of half‐recovery time (*R*_*δ*_ *_T_* = 0.5 at 2.8 ps), the cycle of EIT effect from the start (switching‐off) to half‐recovery state is accomplished within 8.8 ps. During this period, the group delay can also be modulated up to ≈3.3 ps.

**Figure 6:**
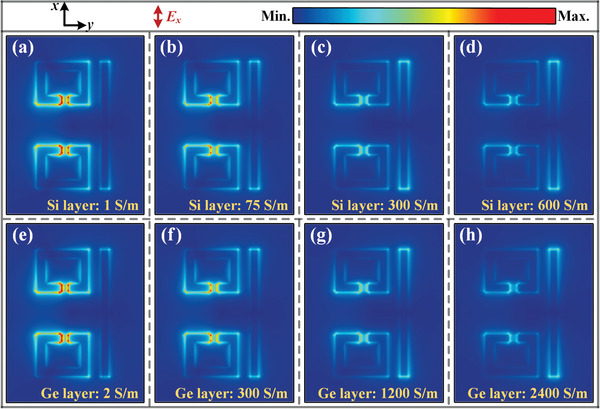
Numerically calculated electric field distributions within one‐unit cell extracted at the EIT resonance frequency. a–d) *E*‐field variations shown in the split gap of the SRRs with a variety of conductivities in the silicon layer, corresponding to the NIR pumped case. e–h) *E*‐field variations shown in the split gap of the SRRs with a variety of conductivities in the germanium layer, corresponding to the NUV pumped case.

From the confined electric near‐fields for EIT resonance presented in **Figure** **6**, one can see that it is feasible to adjust the strength of confined field intensity by tuning the conductivity of both PALs. The field distributions are plotted at the frequency of 0.65 THz representing the maximum EIT resonance. The conductivity of the PALs mainly depends on the photogenerated free carriers, resulting in the screening of near‐field energy in the meta‐atoms and then affecting the THz transmission and group delay. The modeled DC‐conductivity that changes as the photoexcitation fluence is selected by comparing the modulated spectra in Figure S2 (Supporting Information) with that in Figure 2. Indeed, the typical EIT meta‐atoms system proposed in our metadevice consists of a pair of Lorentzian oscillators, whose coupling effect is generally regarded as the interaction between bright and dark modes. By modeling the resonance of quantum transitions, the paradigm can be viewed as a three‐level atomic system involving a dipole‐allowed transition |0〉→|1〉 in the CW and a dipole‐forbidden transition |0〉→|2〉 in the SRR pair. The two pathways of |0〉→|1〉 and |0〉→|1〉→|2〉→|1〉, whose ground state is set as |0〉, interact with each other destructively, accompanied by the appearance of a narrow EIT transparency peak with a suppression of loss. Simultaneously, substantial surface currents are induced in the SRRs, which is characterized by the enhancement of the THz field concentrated at the SRR gaps. The photoactive switching sensitivity is caused by short‐circuiting the capacitive gaps in SRRs, leading to a synchronous annihilation of EIT resonance and an effectively switched‐off modulation. Figure 6a–d presents the *E*‐field distributions as a function of the conductivity change in the PAL II (i.e., 500 nm silicon epilayer). The conductivity of PAL II is 1 S m^−1^ without pumping (according to the information from manufacturers) and increases to 600 S m^−1^ with a saturable modulation depth. As we shall see, the giant field enhancement within the split gaps disappears significantly, accompanied by a switched‐off EIT resonance. In a similar manner, the photoconductivity in PAL I also screens the electric fields in the gaps with relatively higher conductivity values due to its thinner thickness (i.e., 120 nm of noncrystalline Ge) compared to the Si layer.

## 3 Conclusions

In summary, we have proposed and experimentally demonstrated a novel functionality of the active metaphotonic devices, where the EIT meta‐atom hybrid by double photoactive layers showed a pump‐wavelength‐controlled switching behavior at the THz regime. The modulation dynamic of terahertz transmission and group delay spectra exhibited a high sensitivity to the alteration of the optically pumping color from NIR to NUV, resulting in a large half‐recovery time contrast from nanosecond to picosecond scale, with a speed improvement of more than two orders of magnitude. Additionally, by introducing integrating epitaxial Si with amorphous Ge, we have induced an additional degree of freedoms in the penetration depths and trap‐assisted recombination sites in semiconductors. The NIR pump excited the free carriers in Si layer at a sub‐nanosecond relaxation timescale and with a high penetration ratio through Ge layer, whereas the NUV pump is merely injected into Ge layer, showing a sub‐picosecond decay time. In the proposed metaphotonic platform, a normalized transmission resonance modulation exhibits a half‐recovery time of 780 ps with an associated group delay modulation up to 3.3 ps, when pumped by an NIR beam. As for the NUV pumping case, a whole cycle of EIT effect switching with a half‐recovery state is accomplished within 8.8 ps, corresponding to a simultaneous group delay up to 3.3 ps. This metadevice, operating at all‐optically controlled ultrafast speed, can readily be empowered by pump‐color selective manipulation of switching speed from sub‐GHz to several hundred GHz, opening up new avenues for ultrafast terahertz switching photonics. Finally, the spectral scalability is beneficial for designing highly functional and versatile ultrafast optoelectronic devices, and thus further improving the possibility of controlling photonics in unprecedented ways.

## 4 Experimental Section

##### Device Fabrication

The meta‐atoms were fabricated on a commercially available SOS wafer substrate covering a 500 nm thick intrinsic epitaxial Si layer on a 500 µm thick *R*‐plane sapphire. According to the manufacturer, the smoothness of the SOS wafer was ensured to be *Ra* < 0.3 nm by Epi‐polishing and the intrinsic resistivity of the Si layer was 100 Ω cm. First, the hybrid meta‐atoms device was patterned by the well‐designed structure on the Si layer with a standard UV‐lithography technique. A 10 nm chromium adhesion layer and 150 nm gold were sequentially deposited onto the surface by using an electron‐beam evaporator, then followed by a lift‐off process. Next, a 120 nm thick Ge was thermally sputtered onto the prefabricated meta‐atoms with the intrinsic resistivity of 50 Ω cm. The final fabricated double‐PA hybrid meta‐atoms account for an area of 5 × 5 mm^2^.

##### Terahertz Transmission Measurement

Two 1 mm thick ZnTe (110) crystals act as the THz generator and detector. A regenerative amplifier system of spectra physics was employed to provide a 1 kHz repetition laser source with a pulse width of 120 fs (a full‐width at half‐maximum) centered at the wavelength of 800 nm. A linearly polarized pump laser pulse impinged on the hybrid THz modulator side normally with the diameter 5 mm uniformly covering the THz beam spot (≈2.2 mm diameter). Based on the electro‐optic sampling technique through the gating pulses, the THz time‐domain waveforms were recorded in the time domain by moving the translational stage to change the optical path. Then, a standard Fourier transformation of the time‐domain waveforms was performed to obtain the frequency‐dependent THz amplitude. The time delay of optical‐pump THz‐probe was controlled by changing the translational stage of the pumping beam with respect to the THz pulse. Finally, all measured data were carried out by a standard Flourier transformation and normalized by dividing the data of a pure sapphire substrate (*R*‐plane: 500 µm).

##### Electromagnetic Simulation

The full‐field electromagnetic wave responses of the double‐PA hybrid meta‐atoms were determined by the most frequently used finite‐element method (FEM). The incident THz plane wave polarized along the *x*‐direction was propagated along the *z*‐direction within a periodic unit cell whose electric field intensity was set as 1 V m^−1^. For boundary conditions, perfectly matched layers (PMLs) along the *z*‐direction and Floquet boundaries along *x*‐ and *y*‐directions were employed to describe the endlessly repetitive meta‐atoms. The photoconductivity of the Si and Ge layers was optimized to accord with the experimental results. The permittivity of the sapphire was defined as *ε*_Sap_ = 11.7 during simulations according to the THz pulse delay between the cases with and without sapphire substrate. The near‐field distributions were extracted within one period in the *xy*‐plane just above the meta‐atoms.

## Conflict of Interest

The authors declare no conflict of interest.

## Author Contributions

Y.H., J.Y., and M.T. contributed equally to this work. T.J. and Y.H. conceived the idea and designed the research; M.T. and Y.H. fabricated all the samples; Y.H. and X.Z. performed the optical measurement. Y.H., T.J. and J.Y. analyzed the data; Y.H., T.J. and J.Y. co‐wrote the manuscript. All authors discussed and commented on the manuscript.

## Supporting information

Supporting Information
