# Supplementary material for: Pump‐Color Selective Control of Ultrafast All‐Optical Switching Dynamics in Metaphotonic Devices
Source: Adv Sci (Weinh). 2020 Jun 5;7(14):2000799. doi: 10.1002/advs.202000799 (PMC7375251; doi:10.1002/advs.202000799)
Supplement: Supplementary file 1 — Supporting Information [file ADVS-7-2000799-s001.pdf]

Copyright WILEY-VCH Verlag GmbH & Co. KGaA, 69469 Weinheim, Germany, 2020.

Supporting information

Pump-Color Selective Control of Ultrafast All-Optical Switching Dynamics for Metaphotonic Devices

*Yuze Hu, Jie You, Mingyu Tong, Xin Zheng, Zhongjie Xu, Xiangai Cheng and Tian Jiang**

Y. Hu, M. Tong, Prof. Z. Xu, Prof. X. Cheng, Prof. T. Jiang
College of Advanced Interdisciplinary Studies
National University of Defense Technology
Changsha 410073, P.R.China
E-mail: tjiang@nudt.edu.cn
Dr. J. You, Dr. X. Zheng
National Innovation Institute of Defense Technology
Beijing 100010, P.R.China

Supplementary Figures

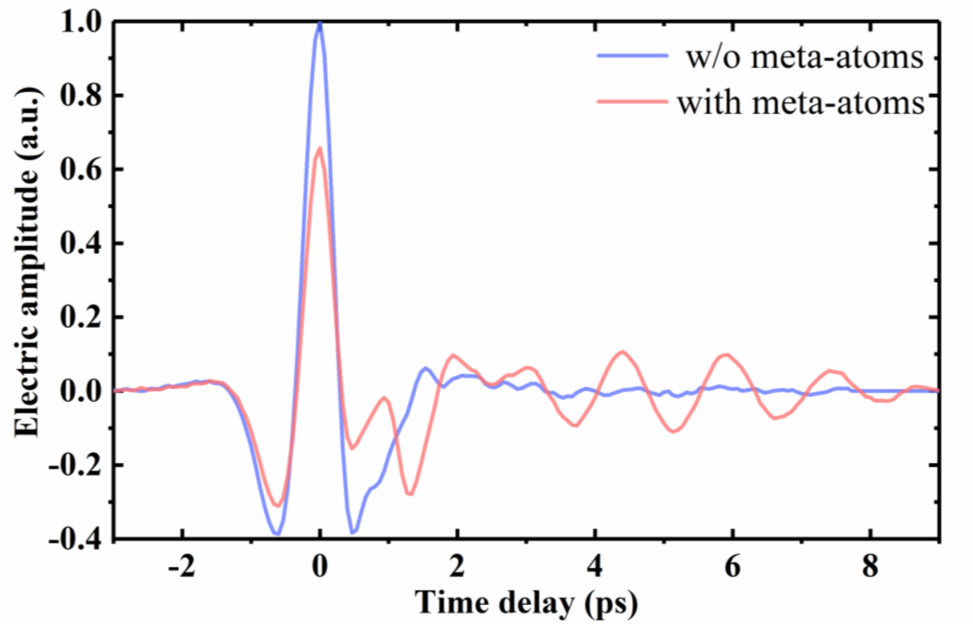

Figure S1. Time-domain transmitted THz pulse waveforms as a function of time delay with and without meta-atoms.

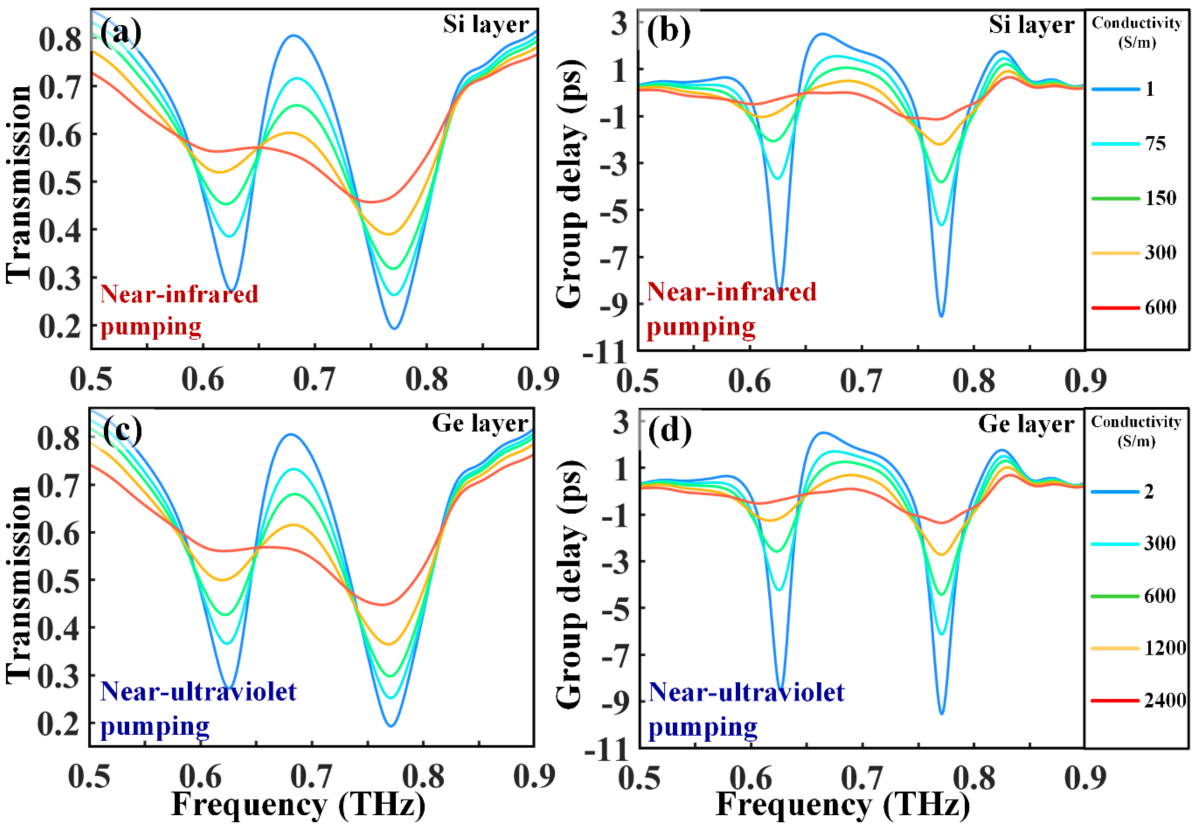

Figure S2. Numerically simulated transmission and group delay spectra by changing the photoconductivity of PALs lying below the EIT meta-atoms. The photoconductivity generated in the silicon layer corresponds to the NIR pumping case, while the change in the germanium layer represents the NUV pumping case. Values of the photoconductivity proportional to the pumping fluence are optimized to match the curves shown in Figure 1 with the same color.

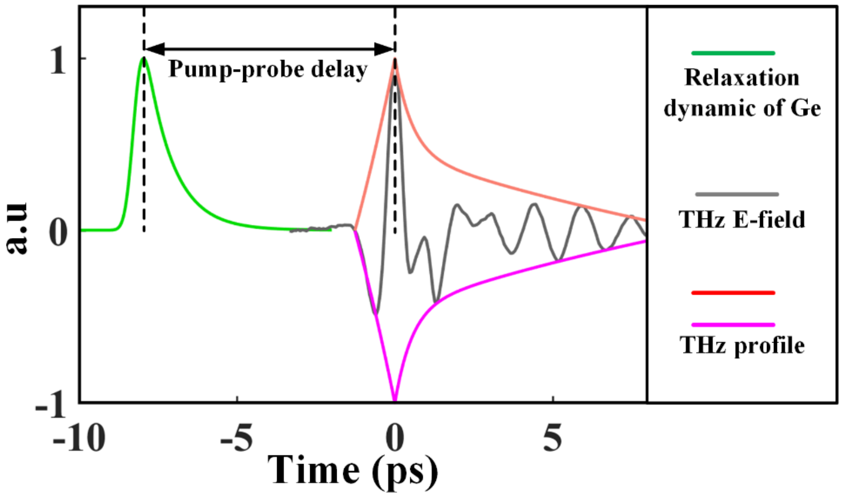

Figure S3. A schematic diagram showing the interaction between photoconductivity of Ge film and THz pulse profile with meta-atoms. The definition of pump-probe delay is clearly defined for the simulation data from Figure S4 to S10.

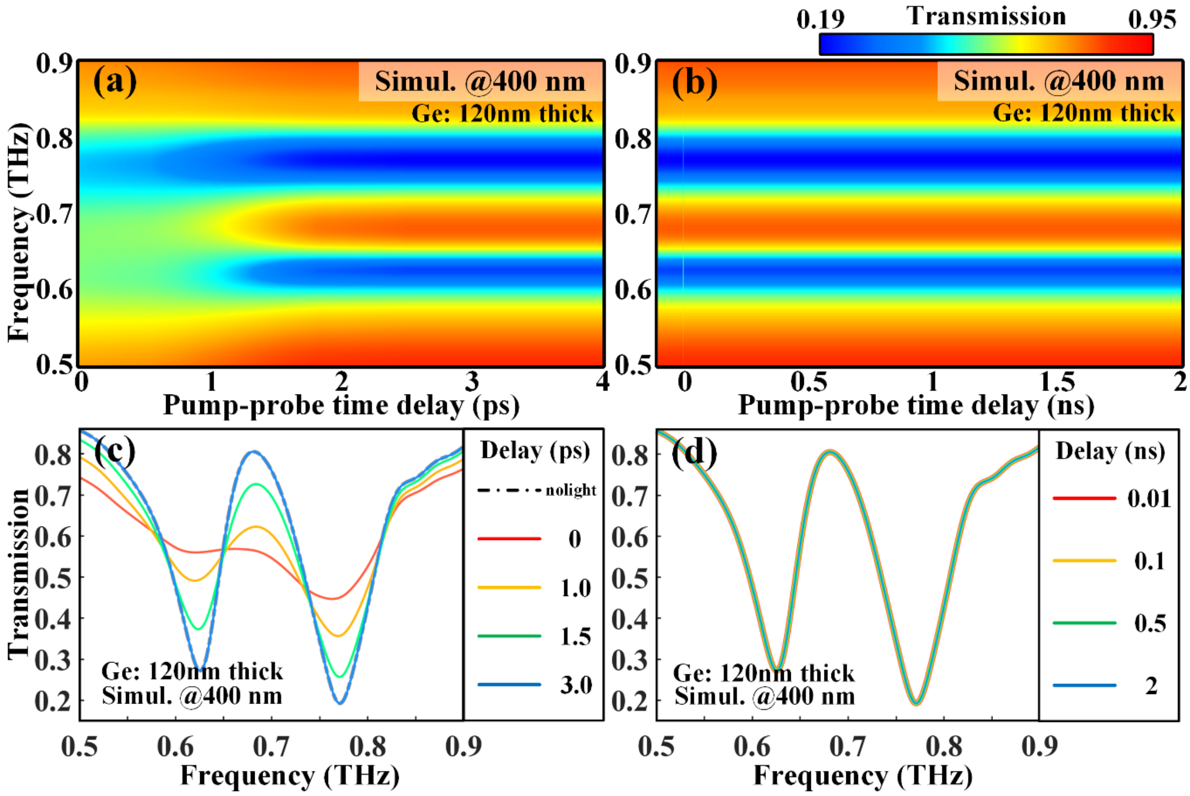

Figure S4. Simulated transient transmission switching dynamics of the double-PA hybrid meta-atoms pumped by the femtosecond pulse centered at 400 nm with the Ge film thickness of 120 nm. a) and c) the evolution dynamic within 4 *ps* which is predominately determined by the Ge film. b) and d) the corresponding dynamic within 2 *ns* determined by the Si film.

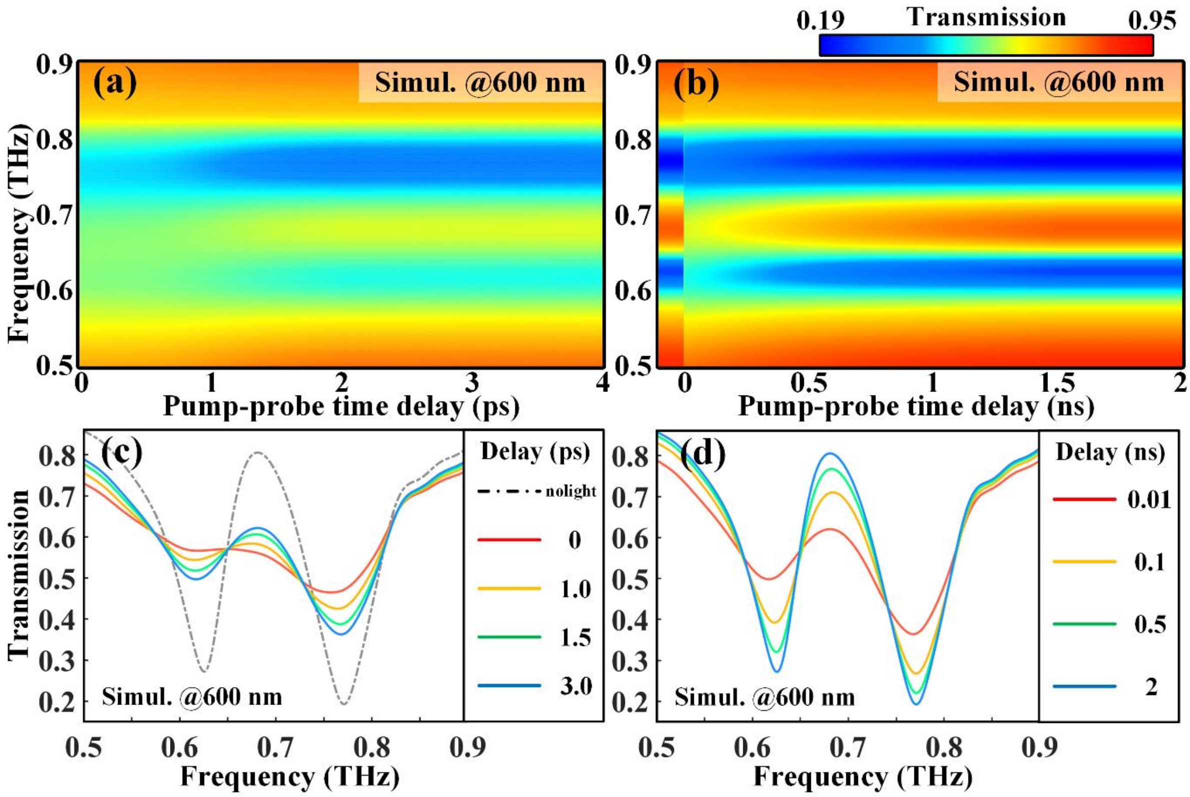

Figure S5. Simulated transient transmission switching dynamics of the double-PA hybrid meta-atoms, pumped by the femtosecond pulse centered at 600 nm, with the Ge film thickness of 120 nm. a) and c) the evolution dynamic within 4 *ps* which is predominately determined by the Ge film. b) and d) the corresponding dynamic within 2 *ns* determined by the Si film.

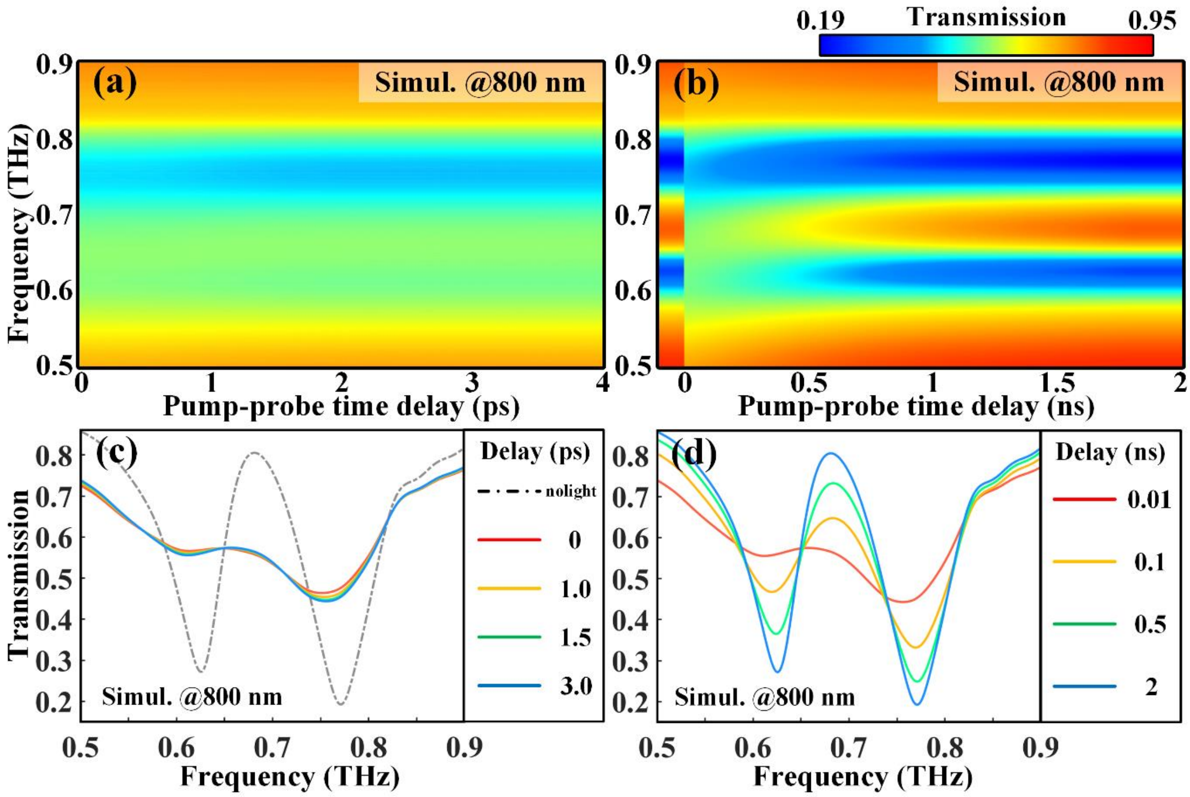

Figure S6. Simulated transient transmission switching dynamics of the double-PA hybrid meta-atoms, pumped by the femtosecond pulse centered at 800 nm, with the Ge film thickness of 120 nm. a) and c) the evolution dynamic within 4 *ps* which is predominately determined by the Ge film. b) and d) the corresponding dynamic within 2 *ns* determined by the Si film.

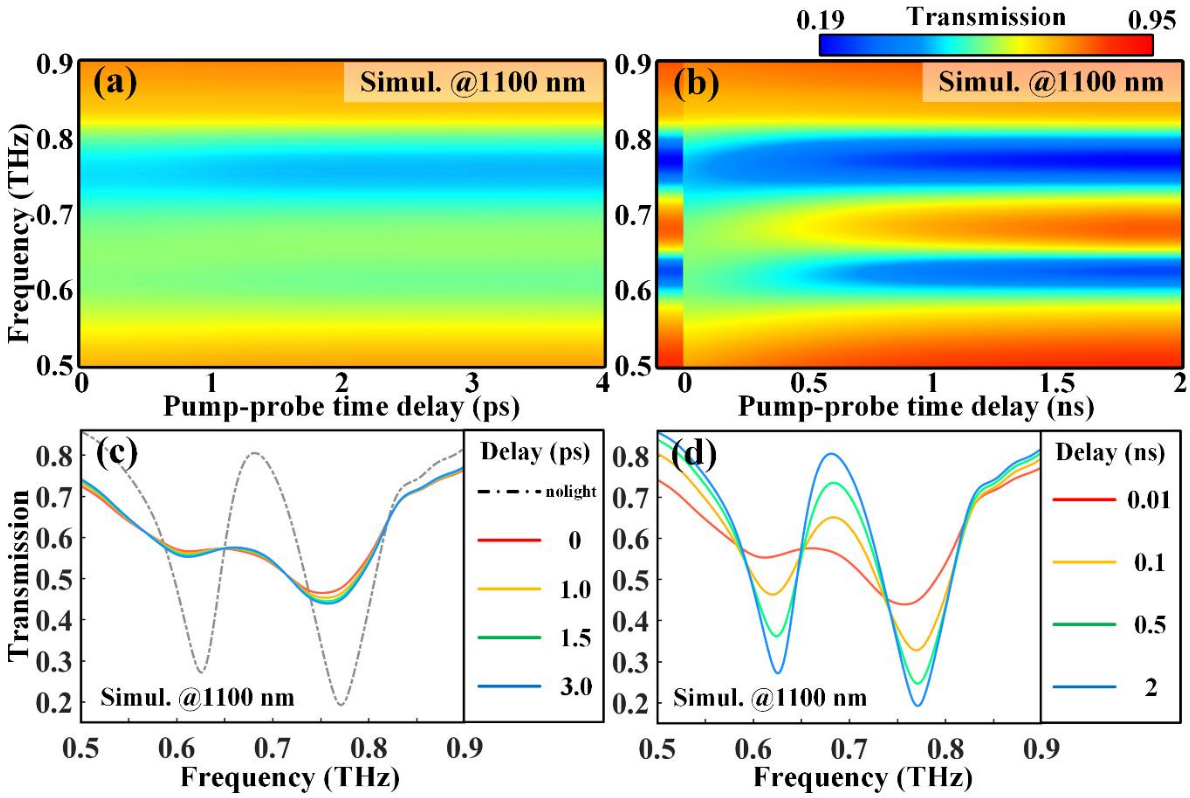

Figure S7. Simulated transient transmission switching dynamics of the double-PA hybrid meta-atoms, pumped by the femtosecond pulse centered at 1100 nm, with the Ge film thickness of 120 nm. a) and c) the evolution dynamic within 4 ps which is predominately determined by the Ge film. b) and d) the corresponding dynamic within 2 *ns* determined by the Si film.

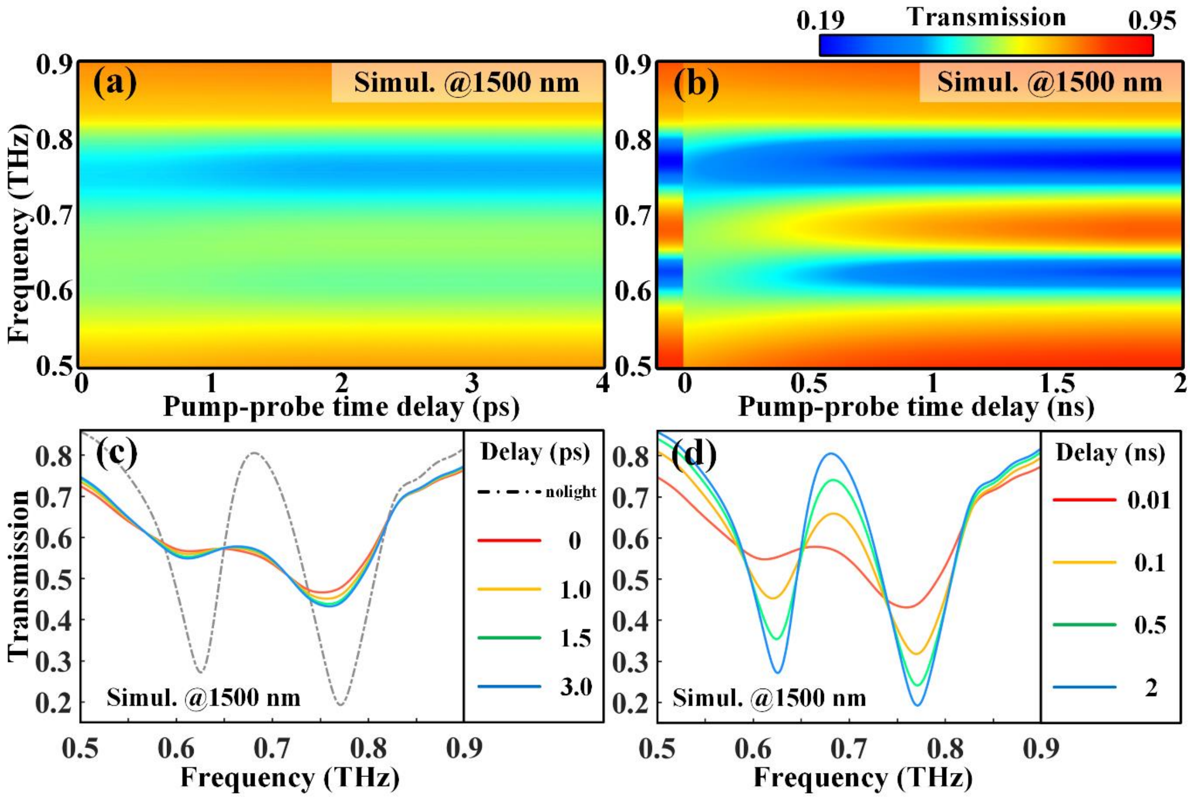

Figure S8. Simulated transient transmission switching dynamics of the double-PA hybrid meta-atoms, pumped by the femtosecond pulse centered at 1500 nm, with the Ge film thickness of 120 nm. a) and c) the evolution dynamic within 4 ps which is predominately determined by the Ge film. b) and d) the corresponding dynamic within 2 *ns* determined by the Si film.

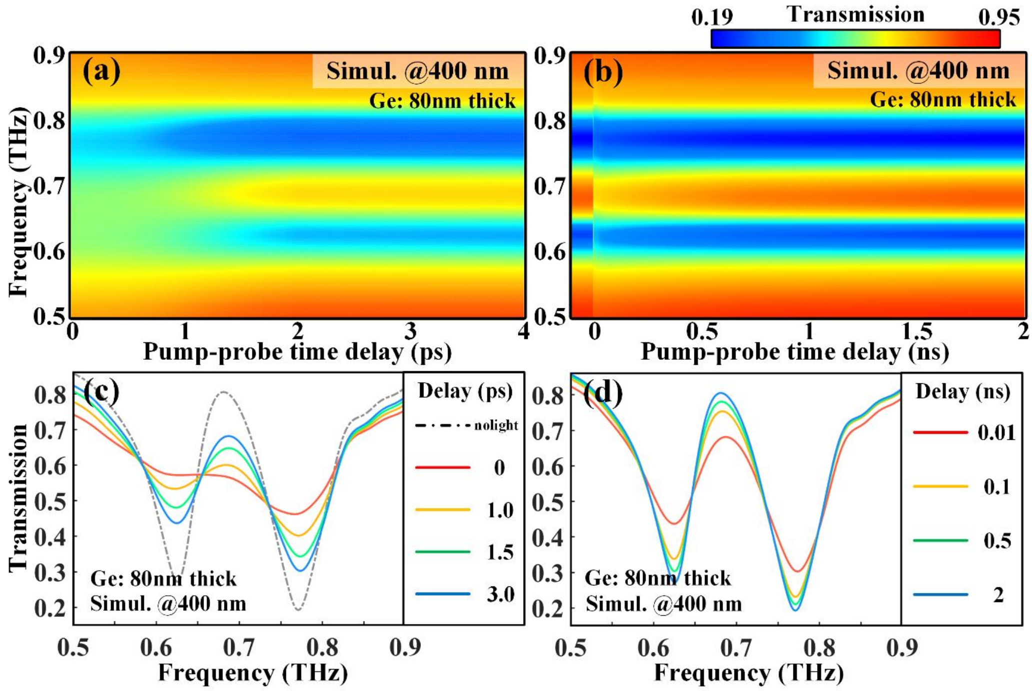

Figure S9. Simulated transient transmission switching dynamics of the double-PA hybrid meta-atoms, pumped by the femtosecond pulse centered at 400 nm, with the Ge film thickness of 80 nm. a) and c) the evolution dynamic within 4 *ps* which is predominately determined by the Ge film. b) and d) the corresponding dynamic within 2 *ns* determined by the Si film.

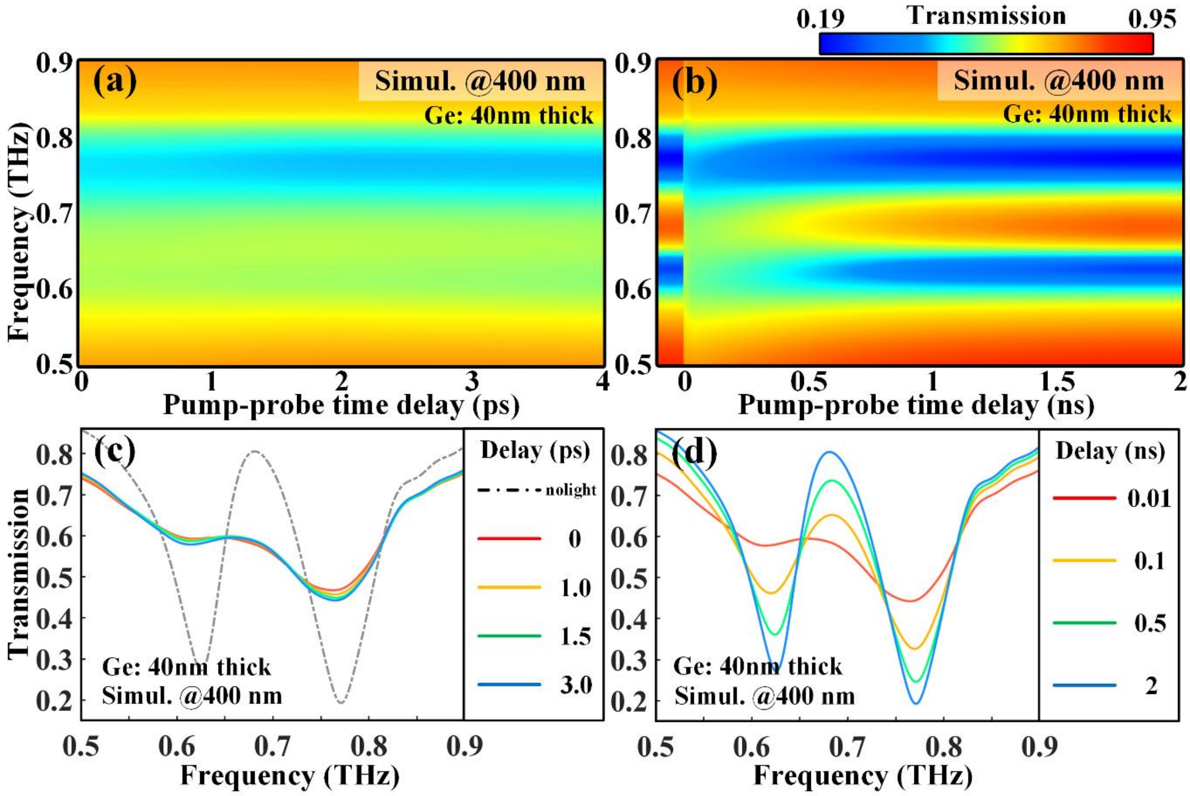

Figure S10. Simulated transient transmission switching dynamics of the double-PA hybrid meta-atoms, pumped by the femtosecond pulse centered at 400 nm, with the Ge film thickness of 40 nm. a) and c) the evolution dynamic within 4 *ps* which is predominately determined by the Ge film. b) and d) the corresponding dynamic within 2 *ns* determined by the Si film.

Supplementary Notes

Supplementary Note S1: Photoconductivity used for transient dynamic simulations

When the double-PA hybrid meta-atoms are pumped by the pump beam, the number of photons absorbed by the Ge and Si films would vary with different pump wavelengths, respectively. Therefore, the photoconductivity of each layer should be determined separately at the interested pump wavelength. Then, the photoconductivity of Ge disappears within several picoseconds due to its fast relaxation time, whereas the corresponding Si photoconductivity can last until more than 1 nanosecond after pumping. Herein, the calculation of transient THz responses is divided into three steps: 1) Determination of the initial effective conductivity of Ge and Si; 2) Calculations of the effective conductivity in each PA layer as a function of time delay; 3) Simulations of THz spectra according to effective conductivity.

We establish a semiempirical model to obtain the ratio $\sigma_{Ge,\lambda}/\sigma_{Si,\lambda}$ according to our experimental results. Since the effective permittivity of Ge and Si at the wavelength from 400 nm to 1500 nm is available, we can readily derive the absorption ratio $A_{Ge,\lambda}/A_{Si,\lambda}$ when illuminated by a beam at the wavelength λ. On the other hand, the photoconductivity can be approximately estimated as:

$$\sigma \propto N_{\mathrm{eh}} \propto \frac{PA}{V\hbar\omega} \propto \frac{FA\lambda}{H}$$

where P is the input power, A is the absorption in the PA layer, V is the PA layer volume, F is the pump fluence, H is the height of the PA layer. As a result, we can obtain the $\sigma_{Ge,\lambda}/\sigma_{Si,\lambda}$

$$\frac{\sigma_{Ge,\lambda_1}}{\sigma_{Si,\lambda_2}} = \frac{F_{Ge,\lambda_1} A_{Ge,\lambda_1} \lambda_1 H_{Si}}{F_{Si,\lambda_2} A_{Si,\lambda_2} \lambda_2 H_{Ge}} \alpha$$

where the coefficient α is needed to be calculated according to our experimental results. If we take $\lambda_1 = 400\ \text{nm}$, then $\sigma_{Ge,\lambda_1} = 2400\ S/m$ and $F_{Ge,\lambda_1} = 1100\ \mu\text{J}/\text{cm}^2$. When λ_2 equals to 800 nm, we can derive $\sigma_{Si,\lambda_2} \approx 600\ S/m$ by utilizing the relation $\sigma = \varepsilon_0 c/d\,(n_{air} + n_{sub})(-\Delta E/E_0)$ and the results in Figure 3a. The fluence illuminated onto the Si layer F_{Si,λ_2} can be easily obtained by taking out the reflection and absorption parts in Ge layer when pumped at 200 uJ/cm^2. Thus, we can get the value of α at this step.

Next, the ratio $\sigma_{Ge,\lambda}/\sigma_{Si,\lambda}$ at any interested wavelength can be retrieved according to the equation:

$$\frac{\sigma_{Ge,\lambda}}{\sigma_{Si,\lambda}} = \frac{F_{Ge,\lambda} A_{Ge,\lambda} H_{Si}}{F_{Si,\lambda} A_{Si,\lambda} H_{Ge}} \alpha$$

It should be noted that the input pump fluence at any wavelength may be changed in order to gain sufficient modulation depth. Once this ratio is fixed, we then try to adjust the conductivity to get modulation depth just up to 100% of the suppression of EIT resonance, leading to the determination of initial effective conductivity in Ge and Si layers.

The effective photoconductivity of Si layer is considered to be decayed exponentially as in Figure 3a, since the THz pulse profile (several picoseconds) is much shorter than the relaxation time of carriers in Si (more than a nanosecond). However, the decay constant of free carriers in Ge film is less than the width of THz pulse profile, as clearly shown in Figure S3. In our experiment, a pump pulse is used to excite the free carriers in Ge film, leading to the photoconductivity in the Ge layer existing only about 2 picoseconds. The overlap between Ge photoconductivity profile and THz profile results in the modulation of EIT effect, and its maximal overlap time is defined as 0 pump-probe time delay corresponding to a maximal effective photoconductivity of Ge film. Subsequently, the effective photoconductivity decreases versus time as the overlap region diminishes gradually.

After the effective photoconductivity of Ge and Si as a function of pump-probe time delay is determined, the THz responses of this metadevice can be calculated on the basis of the

methods described in the section of *Electromagnetic Simulation*. The transient switching-on dynamics by varying the pump wavelength and the thickness of Ge film are unambiguously demonstrated in Figure S4, S5, S6, S7, S8, S9, and S10.

Supplementary Note S2: Switching dynamics influenced by the pump wavelength

Since the newly developed combination of double photoactive layers with metasurfaces in this work is to verify that pump-color selective switching dynamic changed from nanoseconds to picoseconds, it is also necessary to compare the switching dynamics at pump wavelengths covering the absorption band of Ge and Si material. The numerical data covering the wavelength from 400 nm to 1500 nm is vividly illustrated in Figure S4, S5, S6, S7, S8 for 400 nm, 600 nm, 800 nm, 1100 nm, 1500 nm, respectively. As expected from the relaxation dynamics in Ge and Si, the switching dynamic can be classified as two processes: fast process within 4 *ps* and slow process within 2 *ns*. Consistent with our experimental expectation pump by NVU-beam (i.e. 400 nm), the switching dynamic depends solely on the Ge film with ultrafast speed on the time scale several picoseconds (shown in Figure S4), then followed by no evolution process since photons absorbed in Si are negligible. When pumped by the NIR-beam (i.e. 800 nm, 1100 nm, 1500 nm in Figure S6, S7, S8, respectively), the fast switching process exists but is rather small so that its switching effect can be ignorable. Thus, the switching-on dynamics in these cases are almost dependent on the relaxation of free carriers in Si. The mechanism behind is that the penetration length of Ge is longer than the corresponding thickness so that sufficient number of photons is illuminated into the Si layer. The photoconductivity generated in Si layer almost leads to the saturable switching-on state of EIT effect, and thereby the additional small photoconductivity in Ge layer causes little influence on the annihilation of EIT effect. An intriguing phenomenon observed in Figure S5 is that both fast and slow switching-on processes are obvious as pumped by visible light (i.e. 600 nm). The above results indicate that we can actively change the proportion of fast

switching process by varying the pump wavelength in the visible light region, offering a degree of freedom to control ultrafast dynamics for all-optical switching metadevices.

Supplementary Note S3: Switching dynamics influenced by the PA layer thickness

Since the dynamic of proposed metadevice mainly relies on the number of photons penetrating through the Ge layer, we herein systematically investigate the switching dynamics affected by the thickness of Ge layer, as shown in Figure S4, S9, S10. When the pump wavelength is selected as 400 nm, we purposely change the thickness of Ge layer from 120 nm to 40 nm to observe the distinction of switching dynamics. As anticipated, the fast switching process is less dominant as the thickness of Ge layer decreases. It is noticeable that no fast switching process occurs even pumped by NUV beam in the case for the thickness of Ge layer down to 40 nm. Therefore, no fast switching process can be achieved by varying the pump wavelength if the Ge layer is too thin. On the other hand, no slow switching process takes place if the Ge layer is too thick to prevent the penetration of photons through the Ge layer. To conclude, the thickness of Ge layer intimately plays an important role in the appropriateness of realizing pump-color selective ultrafast dynamics in the proposed metadevice.
